# Unveiling the Bacterial Community across the Stomach, Hepatopancreas, Anterior Intestine, and Posterior Intestine of Pacific Whiteleg Shrimp

**DOI:** 10.4014/jmb.2403.03039

**Published:** 2024-04-29

**Authors:** Dhiraj Kumar Chaudhary, Sang-Eon Kim, Hye-Jin Park, Kyoung-Ho Kim

**Affiliations:** 1 Department of Microbiology, Pukyong National University, Busan 48513, Republic of Korea; 2 Division of Marine and Fisheries Life Sciences, Pukyong National University, Busan 48513, Republic of Korea; 3 Korea Institute of Ocean Science and Technology, Busan 49111, Republic of Korea

**Keywords:** Pacific whiteleg shrimp, gut microbiome, bacterial community, gastrointestinal tract, aquaculture

## Abstract

The gastrointestinal (GI) tract of shrimp, which is comprised of the stomach, hepatopancreas, and intestine, houses microbial communities that play crucial roles in immune defense, nutrient absorption, and overall health. While the intestine's microbiome has been well-studied, there has been limited research investigating the stomach and hepatopancreas. The present study addresses this gap by profiling the bacterial community in these interconnected GI segments of Pacific whiteleg shrimp. To this end, shrimp samples were collected from a local aquaculture farm in South Korea, and 16S rRNA gene amplicon sequencing was performed. The results revealed significant variations in bacterial diversity and composition among GI segments. The stomach and hepatopancreas exhibited higher Proteobacteria abundance, while the intestine showed a more diverse microbiome, including Cyanobacteria, Actinobacteria, Bacteroidetes, Firmicutes, Chloroflexi, and Verrucomicrobia. Genera such as *Oceaniovalibus*, *Streptococcus*, *Actibacter*, *Ilumatobacter*, and *Litorilinea* dominated the intestine, while *Salinarimonas*, *Sphingomonas*, and *Oceaniovalibus* prevailed in the stomach and hepatopancreas. It is particularly notable that *Salinarimonas*, which is associated with nitrate reduction and pollutant degradation, was prominent in the hepatopancreas. Overall, this study provides insights into the microbial ecology of the Pacific whiteleg shrimp's GI tract, thus enhancing our understanding of shrimp health with the aim of supporting sustainable aquaculture practices.

Pacific whiteleg shrimp (*Litopenaeus vannamei*) is a major cultured shrimp in the aquaculture industry. This species represents a cornerstone commodity of the global aquaculture system, with a total production of 5.8 million tonnes in 2020 [1]. Asia and Latin America are the predominant regions for shrimp farming, within which China, Thailand, Indonesia, India, Vietnam, Ecuador, Mexico, Brazil, and Honduras are the top shrimp-producing countries [1-3]. Shrimp aquaculture industries are currently experiencing rapid growth and making substantial contributions to economic development [4]. However, the overall robustness of shrimp farming and its proliferation primarily depends on the health status of the shrimp themselves. Any disease outbreak can result in a significant loss to these industries [5]. Therefore, it is considered to be of utmost importance to promote the optimal health and growth of shrimp, which are greatly associated with the microbiome of the shrimp gastrointestinal (GI) tract.

The GI tract of shrimp is composed of foregut (stomach), midgut (hepatopancreas), and hindgut (intestine) [6]. The microbial communities in these three components of the GI tract play a pivotal role in immune defense, nutrient absorption, nutrient digestion, and overall health [7]. The microbiota of the shrimp’s GI tract is related to various health and disease states. Beneficial microbes protect shrimp from pathogens, mediate digestive function and immune response, and provide essential nutrients [7, 8]. Meanwhile, dysbiosis conditions of the GI tract can lead to several diseases, including acute hepatopancreatic necrosis disease, early mortality syndrome, and white feces syndrome [6, 9]. It is paramount to understand the intricacies of the microbiome in the GI tract of shrimp to advance the current knowledge of shrimp health and aquaculture practices.

Among the three sections of the GI tract, the intestine has been the subject of the most research, and the intestine’s microbial structure has been the best profiled [5, 6, 10]. The intestinal microbial community has attracted considerable attention because of its diverse influence on host physiology and metabolism [3, 7]. The intestinal microbiome of shrimp not only supports digestion and nutrient absorption but also plays a crucial role in immune system modulation [11]. Studies have also shown that the microbial communities in the anterior and posterior segments of the intestine of aquatic invertebrates are distinct from each other and may perform diverse functions [12, 13]. However, there have been few studies examining the microbiomes of the anterior and posterior segments of shrimp intestine. The hepatopancreas is primarily responsible for digestive enzyme production, nutrient metabolism, and regulating host innate immunity. The numerous types of bacteria residing in the hepatopancreas support various developmental and physiological functions of shrimp [9, 14]. Although it has to this point been relatively understudied in shrimp, the stomach likely harbors a distinct microbial community that aids in digestion and nutrient processing [15, 16].

The colonization of microorganisms in the GI tract of whiteleg shrimp begins during the nauplius 5 stage and continues to gradually develop in the entire GI tract throughout different life stages [17]. Bacterial genera that are commonly reported in the intestinal segment of GI tract include *Vibrio*, *Streptococcus*, *Sphingomonas*, *Shewanella*, *Ruegeria*, *Pseudomonas*, *Paracoccus*, *Lactobacillus*, *Flavobacterium*, *Clostridium*, *Bacillus*, and *Acinetobacter* [18-20]. Numerous bacteria have been also reported from hepatopancreas segment, including *Pantoea*, *Ochrobactrum*, *Octadecabacter*, *Phenylobacterium*, *Shigella*, *Stenotrophomonas*, and *Vibrio* [9, 20, 21]. There have only been a few studies examining the microbiota in the stomach of whiteleg shrimp. *Vibrio*, *Isoptericola*, *Rhodobacteraceae*, *Photobacterium*, and *Pseudoalteromonas* have been documented in the stomach of shrimp [16, 22]. Various studies have noted colonization of *Vibrio* strains in all three segments of GI tract of shrimp. However, previous investigations have shown that the prevalence of several other key bacteria differed between the three segments (*i.e.*, intestine, hepatopancreas, and stomach) [9, 14, 16, 22]. For example, Cornejo-Granados *et al*. (2017) illustrated the difference in bacterial diversity between intestine and hepatopancreas by showing that *Vibrio shilonii*, *Faecalibacterium prausnitzii*, and *Aeromonas taiwanensis* were detected in the intestine whereas *Pantoea agglomerans* and *Photobacterium angustum* were observed in the hepatopancreas [9]. Imaizumi *et al*. (2021) showed a higher bacterial diversity in the stomach compared to that in the intestine [16]. Altogether, these microbial communities have the potential to contribute to the digestive process and help maintain overall health among shrimp.

Despite the significant importance of the shrimp gut microbiome, there have been few comprehensive studies examining the bacterial communities within each of the intestine (anterior and posterior segments), hepatopancreas, and stomach. Existing studies have mainly focused on specific regions of the GI tract, thus leaving a critical knowledge gap regarding the holistic microbial ecology among these interconnected segments. Therefore, the present study intended to address this gap by analyzing an in-depth bacterial community structure inhabiting the anterior and posterior intestine, hepatopancreas, and stomach of Pacific whiteleg shrimp. By profiling the taxonomic composition, diversity, and potential function of key bacteria, we aimed to unravel the intricate relationships between the shrimp host and its resident bacteria. The findings obtained herein hold great promise for enhancing the current understanding of the microbiome in various segments of GI tract of Pacific whiteleg shrimp, and may contribute to the sustainable management of the shrimp aquaculture system.

## Materials and Methods

### Shrimp Sample Collection and Dissection

Pacific whiteleg shrimp (*Litopenaeus vannamei*) were collected from a local aquaculture farm located in Goseong, Gyeongsangnam-do, Republic of Korea. Upon collection, the shrimp were immediately frozen, transported to the laboratory, and stored at -80°C until further analysis. In total, nine healthy shrimp (labelled as LA1-LA9) were randomly selected for dissection and had their weights and sizes measured. The lengths of the shrimp ranged between 8.9 cm to 13.8 cm while the weights thereof ranged between 3.2 g to 14.0 g. The shrimp stomach, hepatopancreas, anterior intestine, and posterior intestine were aseptically dissected from each specimen and immediately used for DNA extraction.

### 16S rRNA Gene Amplicon Sequencing

Total DNA was extracted separately from each sample of stomach, hepatopancreas, anterior intestine, and posterior intestine using the E.Z.N.A. Soil DNA extraction kit (catalogue no. D5625-01; Omega Bio-Tek, USA) following the manufacturer's protocol. DNA concentration was measured using a Nanodrop spectrophotometer (DS-11 FX Series, DeNovix), and DNA integrity was examined using agarose gel electrophoresis. Barcode primers were designed to distinguish each sample by attaching 12 bp different nucleotide sequences to the existing primer set 319F (5'-ACTCCTACGGGAGGCAGCAG-3') and 806R (5'-GGACTACHVGGGTWTCTAAT-3'). The V3-V4 region of the 16S rRNA gene was amplified using these primer sets (Table S1). The PCR reaction was formulated using AccuPower PCR PreMix (Bioneer, Republic of Korea). The PCR amplification was carried out in a thermal cycler (SC200, Kyratec, Australia) under the following cycling conditions: pre-denaturation at 98°C for 60 s; 35 cycles of denaturation at 98°C for 30 s, annealing at 58°C for 30 s, and extension at 72°C for 30 s; and the final extension at 72°C for 60 s. The amplified product was then purified using a BioFact PCR Purification solution kit (Biofact, Republic of Korea). After purification, the concentration of each purified product was determined using a Qubit Fluorometer (Thermo Fisher Scientific, USA). The equimolar concentrations of the purified amplicons were pooled before being used to prepare sequencing libraries using the TrueSeq Amplicon library construction kit (Illumina, USA) according to the manufacturer's instructions. Finally, the sequencing was conducted using Illumina Miseq Platform (PE300) at Macrogen Korea Inc.

### Bioinformatics and Data Analysis

The raw sequence data were processed and analyzed by Quantitative Insights Into Microbial Ecology 2 (QIIME 2, version 2023.9) [23]. The initial raw sequences were demultiplexed and quality filtered using the q2‐demux and q2-dada2 plugins. [24]. Samples with low quality reads were excluded, as a result of which only the qualified data obtained from 27 samples were further analyzed. The obtained amplicon sequence variants (ASVs) were aligned with mafft and used to generate a phylogeny with fasttree2 using the q2‐alignment and q2‐phylogeny plugins. Alpha‐diversity metrics [25], beta diversity metrics [26], and Principle Coordinate Analysis (PCoA) were determined by q2‐diversity. Taxonomic assignment to ASVs was conducted using the q2‐feature‐classifier [27] against the Greengenes 13_8 database with a 99% similarity threshold [28]. The demultiplexed sequence data were deposited in NCBI GenBank under the BioProject accession numbers PRJNA1082065.

Further analyses and data visualization were conducted on the Microbiome Analyst platform, which is a statistical, functional interpretation, visualization, and meta-analysis tool for microbiome data that works on the basis of R packages [29]. The ASV table, metadata file, taxonomy file, and phylogenetic tree obtained from QIIME 2 analysis were uploaded into the Marker Data Profiling tool of Microbiome Analyst platform. Data filtering was performed with the default parameters. All samples were rarified to the minimum library size and normalized using the total sum scaling (TSS) technique. Alpha diversity was computed using the observed species, Shannon, ACE, and Chao1 indexes with *t*-test or ANOVA analysis. Beta diversity was visualized according to the PCoA ordination method using the Unweighted UniFrac Distance, and PERMANOVA was used for statistical analysis. Dendrograms were generated using the Bray-Curtis Index at the feature level. Pattern search was performed using the Pearson r at the phylum level. Linear Discriminant Analysis (LDA) Effect Size (LEfSe) analysis was conducted to explore the significant differential abundance of phylum, and a heat tree was generated using a *p*-value cutoff set to 0.05 and a log LDA score of 2.0. The correlation network analysis was performed using Sparse Estimation of Correlations Among Microbiomes (SECOM) algorithm with the *p*-value threshold set to 0.05 and the correlation threshold set to 0.3.

## Results

### Bacterial Diversity Measurement

**Fig. 1:**
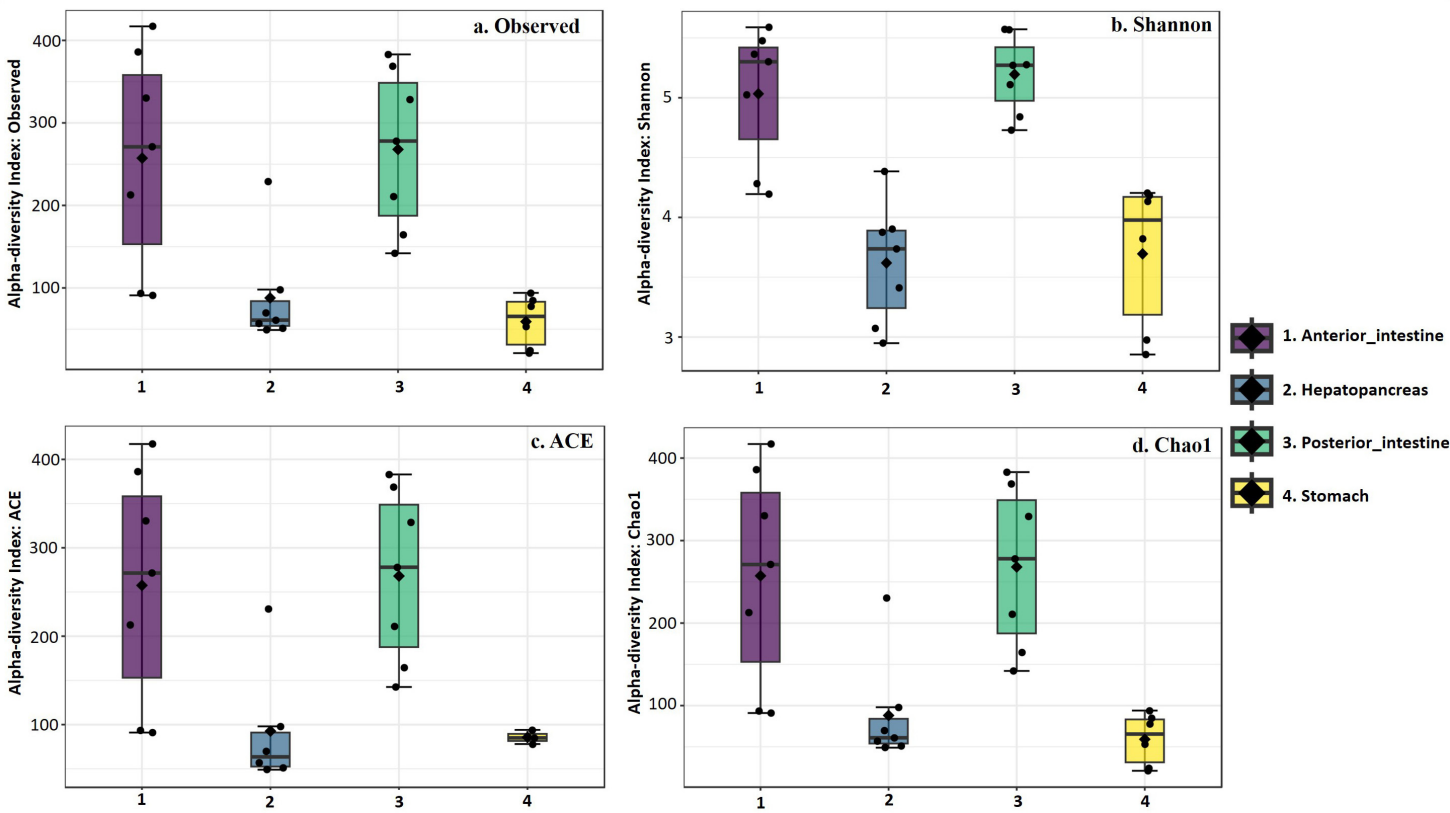
Alpha diversity indexes of bacterial community of whiteleg shrimp across the stomach, hepatopancreas, anterior intestine, and posterior intestine.

The alpha diversity was measured by determining observed ASVs, Shannon, ACE, and Chao1 indexes. At the feature level, all the alpha diversity indexes showed significant differences between shrimp stomach, hepatopancreas, anterior intestine, and posterior intestine (*p* < 0.05). The observed index was highest in anterior intestine (93.0–417.0) and lowest in stomach (21.0–94.0) samples. Shannon index showed significantly higher species richness in both anterior and posterior intestine compared to both hepatopancreas and stomach (*p* < 0.05). The ACE and Chao1 indexes also showed similar trends, resulting in the finding of higher species richness in the samples collected from anterior and posteriors intestine. The samples Bac_A_LA7 (anterior intestine) showed higher diversity indexes, while sample Bac_S_LA5 (stomach) exhibited lower diversity indexes. Overall, the alpha diversity indexes indicated more diversity among the bacterial community in the anterior intestine of shrimp and lower diversity among the bacterial community in the stomach of shrimp (Table S2 and Fig. 1).

**Fig. 2:**
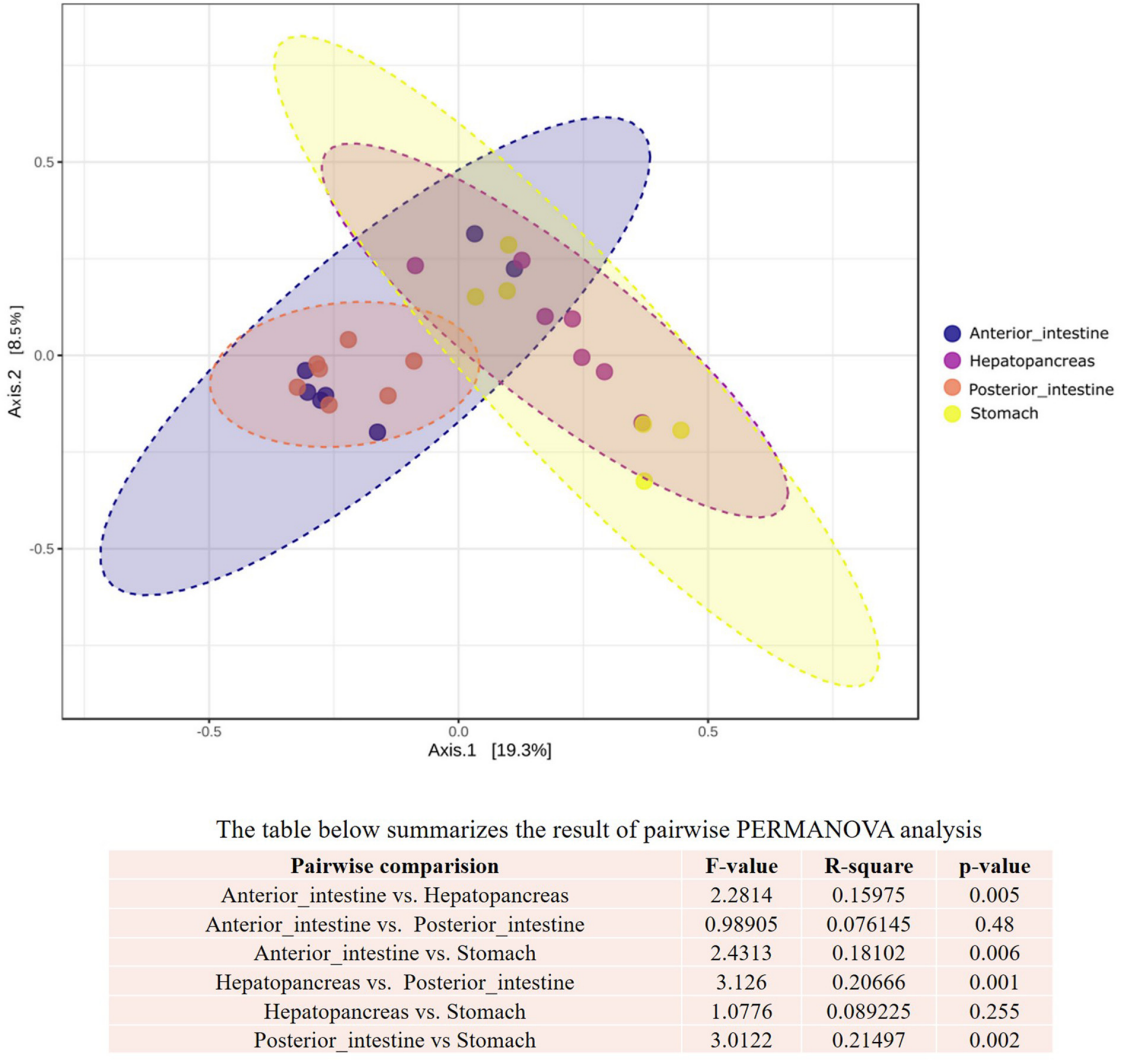
Principal coordinate analysis (PCoA) of beta diversity of bacterial community based on Unweighted Unifrac distance matrix. Statistical analysis was performed using PERMANOVA.

Beta diversity analysis, which was performed using Unweighted UniFrac Distance, showed significant bacterial variation among the stomach, hepatopancreas, and intestines (anterior and posterior) (*p* < 0.05). The PCoA estimated a total variation of 27.8% (axis 1 explained by 19.3% and axis 2 explained by 8.5%). Intestinal samples were clearly distinguished from the hepatopancreas and stomach samples, thus suggesting marked differences in their bacterial composition. However, samples from anterior and posterior intestines overlapped together, and samples from hepatopancreas were clustered within the stomach (Fig. 2). Pairwise PERMANOVA analysis reported a significant difference in the bacterial community structure between anterior intestine vs. hepatopancreas (R^2^ = 0.16, *p* < 0.05), anterior intestine vs. stomach (R^2^ = 0.18, *p* < 0.05), posterior intestine vs. hepatopancreas (R^2^ = 0.20, *p* < 0.05), and posterior intestine vs. stomach (R^2^ = 0.21, *p* < 0.05). By contrast, there were no significant differences observed between the anterior intestine and the posterior intestine (R^2^ = 0.08, *p* > 0.05) or between the stomach and the hepatopancreas (R^2^ = 0.09, *p* > 0.05). Altogether, the bacterial diversity measurement showed prominent variations in bacterial community structure among different fractions of the GI tract of whiteleg shrimp.

### Bacterial Community Composition

**Fig. 3:**
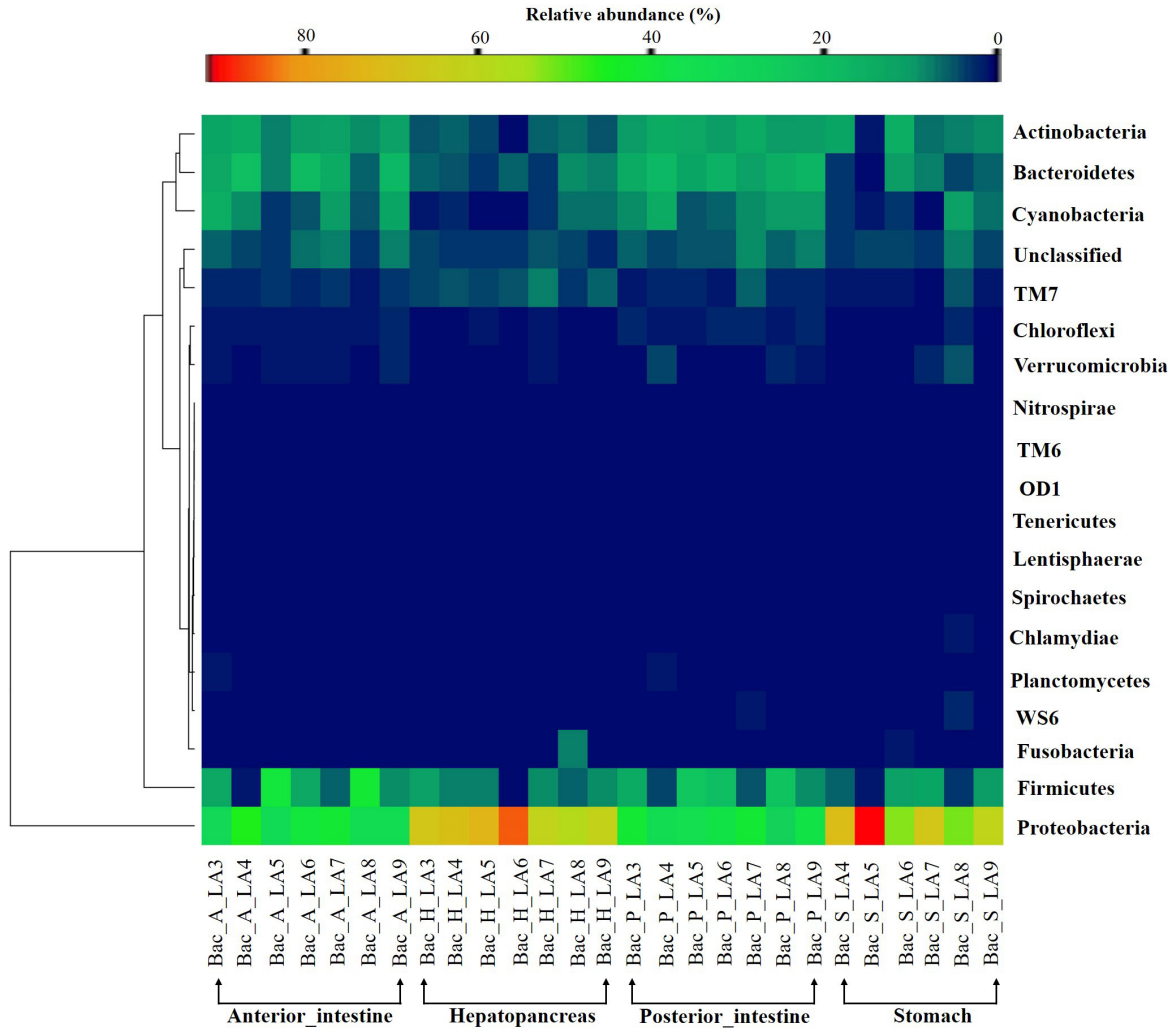
Heat map of predominant phyla in different segments of gastrointestinal tract of whiteleg shrimp.

The bacterial compositions found in all the segments of the GI tract of whiteleg shrimp revealed Proteobacteria as the major phylum, as it accounted for a range of 29%–91% of all bacteria. Hepatopancreas and stomach segments exhibited higher (51.1%–91.0%) abundance of Proteobacteria compared to anterior and posterior intestine (29.1%–45.9%) (Fig. 3). Other higher rank phyla included Cyanobacteria, Actinobacteria, Bacteroidetes, Firmicutes, and Verrucomicrobia. In addition, these phyla were relatively more dominant in the intestine compared to hepatopancreas and stomach (Table S3). The phylum Fusobacteria was prominently detected in the sample Bac_H_LA8 (7.5% relative abundance) of the hepatopancreas and in the sample Bac_S_LA6 (1.3% relative abundance) of the stomach, whereas this phylum remained subordinate in other samples.

**Fig. 4:**
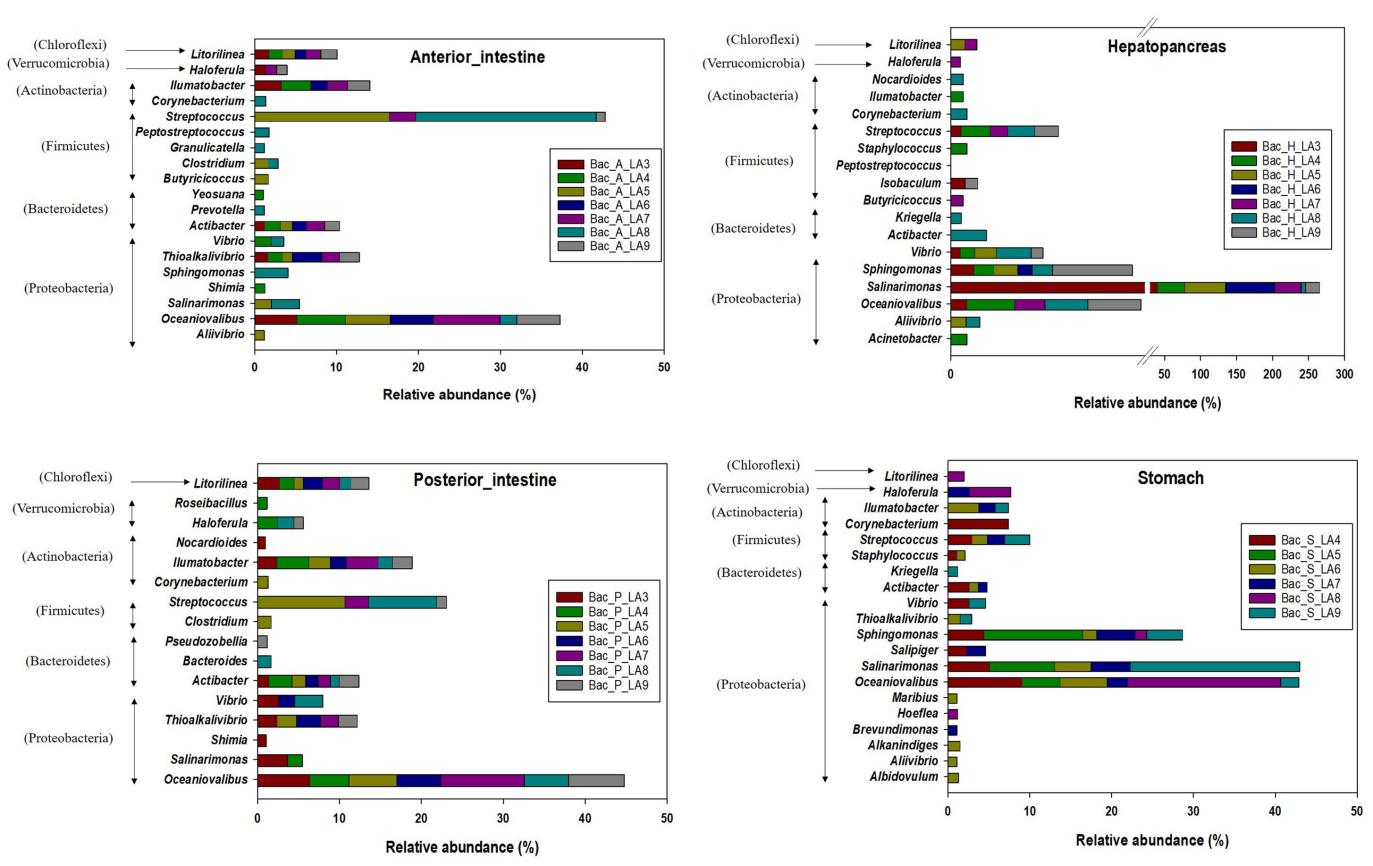
Major genera in stomach, hepatopancreas, anterior intestine, and posterior intestine of whiteleg shrimp. The genus affiliated to respective phylum are represented in parentheses.

The genus level analysis also showed a clear difference in the bacterial composition of stomach, hepatopancreas, anterior intestine, and posterior intestine (Fig. 4). The key genera in the anterior intestine and the posterior intestine were *Actibacter*, *Ilumatobacter*, *Litorilinea* *Oceaniovalibus*, and *Streptococcus*, while the predominant genera in the hepatopancreas and the stomach were *Salinarimonas*, *Sphingomonas*, and *Oceaniovalibus*. The genus *Salinarimonas* was present in notably higher proportions (6.0%–67.8%) in the hepatopancreas segment. *Oceaniovalibus* appeared in all the sections of the GI tract of whiteleg shrimp, ranging from proportions of 2.0% to 18.7%, and it was observed at relatively higher proportions in the stomach (Table S4). Other notable genera found in the gut of whiteleg shrimp included *Vibrio*, *Thioalkalivibrio*, *Aliivibrio*, *Corynebacterium*, and *Haloferula*.

### Difference in the Bacterial Community Structure and Correlation Network Analysis

**Fig. 5:**
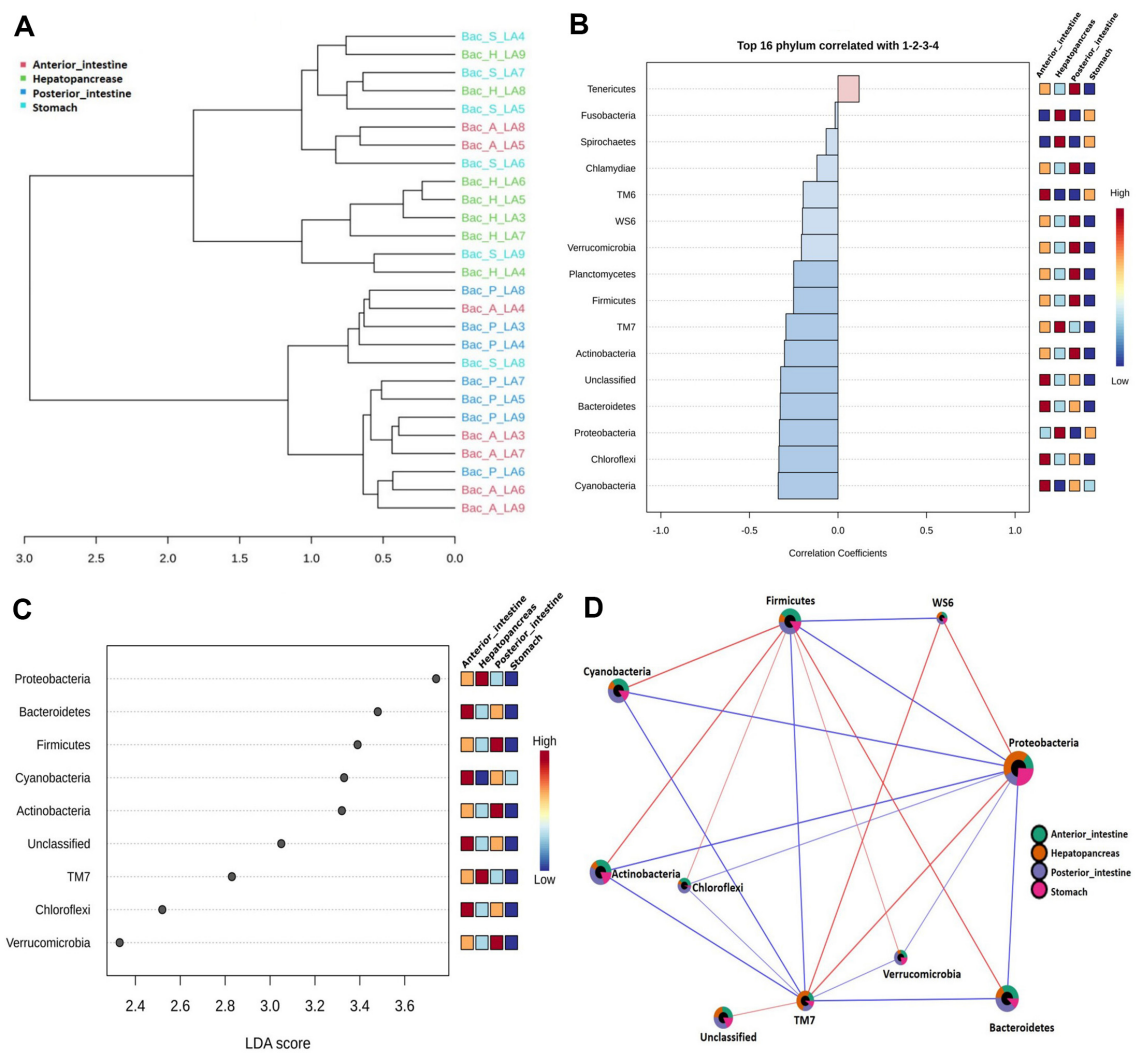
Differential bacterial community structure and correlation network analysis. (**A**) Dendrogram showing hierarchical clustering of shrimp's gastrointestinal (GI) tract samples based on bacterial community structure variation. (**B**) Correlation of top phyla in various segments of GI tract. (**C**) Differential abundant phyla as indicated by LEfSe analysis. The color gradient reflects variations in high to low abundance of the phyla in the related segment of the GI tract. (**D**) Correlation network analysis at the phylum level. Each node indicates a phylum, and its size is determined by the frequency of connections to other phylum. Various colors in the node represent the proportions of GI tract samples (anterior_intestine, hepatopancreas, posterior_intestine, and stomach). The thickness of the edge is equivalent to the correlation coefficients. Red edges indicate positive correlations and blue edges indicate negative correlations.

The dendrogram analysis showed that most of the samples from the anterior and posterior intestines clustered together. Sample-dependent clustering was also observed with the hepatopancreas samples. However, clustering of the stomach samples exhibited different patterns, and they were scattered with various other samples (Fig. 5A). Pattern search analysis reported that Bacteroidetes, TM6, Chloroflexi, and Cyanobacteria were significantly higher in the anterior intestine; Tenericutes, Chlamydiae, WS6, Verrucomicrobia, Planctomycetes, Firmicutes, and Actinobacteria were significantly higher in the posterior intestine; and Spirochaetes, TM7, Proteobacteria, and Fusobacteria were significantly higher in both the hepatopancreas and the stomach (Fig. 5B). The effects of various segments of the GI tract of whiteleg shrimp on the distribution of dominant phyla were examined through LEfSe analysis (Fig. 5C). LEfSe analysis revealed a significant differential abundant feature of phyla in various samples collected from the stomach, hepatopancreas, anterior intestine, and posterior intestine (*p* < 0.05). Bacteroidetes, Chloroflexi, and Cyanobacteria were significantly dominant in the anterior intestine (*p* < 0.05, LDA score 2.5–3.5), while Verrucomicrobia, Firmicutes, and Actinobacteria showed higher effect sizes in the samples collected from the posterior intestine (*p* < 0.05, LDA score 2.3–3.4). Moreover, Proteobacteria and TM 7 were significantly influenced in the hepatopancreas, where they revealed higher abundance (*p* < 0.05, LDA score 2.8–3.7), while no significant differential abundant feature was detected from the stomach samples. Lastly, the correlation network analysis showed significant associations among the bacterial phyla found in various segments of GI tract of whiteleg shrimp (Fig. 5D). Nine phyla—*i.e.*, Proteobacteria, Bacteroidetes, Verrucomicrobia, TM7, Chloroflexi, Actinobacteria, Cyanobacteria, Firmicutes, and WS6—were found to have either positive or negative correlations with each other. Proteobacteria displayed positive correlations with both WS6 and TM7, with correlation values ranging between 0.65 to 0.13, along with negative correlations with Bacteroidetes, Firmicutes, Verrucomicrobia, Chloroflexi, Actinobacteria, and Cyanobacteria, with correlation values ranging between -0.59 to -0.23 (Table S5).

## Discussion

Shrimp GI microbial ecosystems play crucial roles in protecting from pathogens, mediating digestive function, maintaining immune response, and providing essential nutrients in the host [7, 8]. Disturbances in the gut microbiota may cause disease conditions, which may in turn lead to significant economic loss [9]. Therefore, understanding the gut microbiota of shrimp is a prerequisite for achieving sustainable shrimp health and aquaculture practices. However, most studies examining the gut microbiota of shrimp have been limited to investigations of the intestine segment alone [5, 6, 10]. The present study took a more comprehensive approach by exploring the microbiota of different segments (stomach, hepatopancreas, anterior intestine, and posterior intestine) of the GI tract of whiteleg shrimp.

Both the alpha and beta diversities data showed that the gut microbiota of whiteleg shrimp varied based on where among the different segments of the GI tract the samples had been collected from. Alpha diversity found higher bacterial diversity in the anterior intestine and lower bacterial diversity in the stomach segment. Beta diversity exhibited a clear separation in the microbial community of the intestine from those in the hepatopancreas and the stomach. Garibay-Valdez *et al*. (2021) reported a similar trend, as they observed a marked difference in the microbial communities among foregut, midgut, and hindgut [6]. Another study also previously reported observing significantly less diverse microbes in the hepatopancreas compared to the intestine [9]. By contrast, a previous study estimated a significantly higher microbial diversity in the stomach compared to that in the midgut [16]. These observations suggest that whiteleg shrimp may contain a unique ecological niche in different segments of the GI tract which may aid in the proliferation of different types of microbes. Each segment of the GI tract has unique environmental conditions, such as oxygen levels, nutrient availability, and physical characteristics [22, 30]. These conditions may favor the growth of specific types of microorganisms. As an example, the anaerobic conditions in the intestine might support a different microbial community than the more oxygenated environment in the stomach. The particular host immune system, digestive enzymes, nutrient availability, and microbial interaction may allow different microorganisms to establish themselves and persist in each segment of GI tract [31, 32]. Overall, the variation in the bacterial diversity observed in this study among the stomach, hepatopancreas, and intestine of shrimp can be attributed to a combination of anatomical, physiological, and environmental factors [15, 33].

The relative abundance of bacterial composition showed predominance of phylum Proteobacteria in all segments of the GI tract. Proteobacteria has been previously identified as a dominant phylum in shrimp [9, 34]. The results of both pattern search and LEfSe analyses showed that members of the Proteobacteria were found in significantly higher proportions in the stomach and the hepatopancreas compared to the anterior and posterior intestine. Stomach and hepatopancreas are considered to be vital organs in the immune and digestive systems wherein digestive enzymes and immune molecules are secreted [16, 35]. The higher relative abundance of Proteobacteria in the stomach and the hepatopancreas may be associated with their immunological and digestive functions [36, 37]. It has also been speculated that the proteobacterial community reflects the health state of the host, and an increase in its abundance may indicate a potential risk of disease [38]. Further, the phyla Spirochaetes and Fusobacteria were found at significantly increased levels in the hepatopancreas and the stomach. Both these phyla have previously been detected in the hepatopancreas and intestinal sections [9, 39]; however, there have been few reports on the presence of Spirochaetes and Fusobacteria in the stomach. This observation in this study may broaden the available information on the presence of unexplored types of microbiota in the stomach segment of the GI tract of shrimp. Spirochaetes are potentially involved in lignocellulose degradation and nitrogen fixation [40]. Fusobacteria mediates the fermentation of carbohydrates, amino acids, and peptides, and it exhibits a symbiotic relationship with the host leading to better digestive ability [34]. However, research has also shown that Fusobacteria are correlated with the white spot syndrome virus infection in shrimp, thus reflecting a potential pathogenic bacterium [15].

The bacterial profiles of anterior and posterior intestine revealed significantly higher abundance of Cyanobacteria, Actinobacteria, Bacteroidetes, Firmicutes, Chloroflexi, and Verrucomicrobia than those found in the stomach and the hepatopancreas. Members of these phyla have been widely reported in the intestinal samples of whiteleg shrimp [9, 38, 41]. Cyanobacteria may possess antibacterial properties against pathogenic microorganisms, thus pointing to its ability to eradicate pathogens [42]. However, harmful Cyanobacteria may cause eutrophication in the rearing water and release toxins, thus threatening shrimp health [43]. Actinobacteria are promising resources for probiotics and antibiotics, and they play a crucial role in maintaining homeostasis [44, 45]. Firmicutes and Bacteroidetes are both involved in the fermentation process, and they each help provide various nutrients to the host. [38]. The phylum Chloroflexi is able to mediate photosynthesis under anaerobic conditions [38]. It has also been documented that the dominance of Chloroflexi is highly associated with white feces syndrome in shrimp [46]. Increased abundance of Verrucomicrobia is associated with improved metabolic function and an enhanced mucus layer of the host [47]. The differential analysis of bacterial composition reported that Bacteroidetes, Chloroflexi, and Cyanobacteria were comparatively higher in the anterior intestine, while Verrucomicrobia, Firmicutes, and Actinobacteria were relatively enriched in the posterior intestine. Further, the dominant phyla showed either positive or negative networking with each other. The results of correlation network analysis revealed networking within nine phyla. For instance, Proteobacteria correlated negatively with the Bacteroidetes, Firmicutes, Verrucomicrobia, Chloroflexi, Actinobacteria, and Cyanobacteria. This observation also aligns with the bacterial composition data in which we found higher proteobacterial community suppressing other major phyla in the stomach and hepatopancreas segments. These results suggest that higher abundance of one group of bacteria substantially influences another group of bacteria in different segments of the GI tract. Bacteria within bacterial communities compete with each other for habitat and the nutrients in the gut, which inhibits certain bacterial groups. These mechanisms also support the removal of pathogenic bacteria from the gut, thus allowing for a good health status to be maintained in the shrimp [6].

At the genus level, a unique set of genera were found to prevail in each segment of the GI tract of whiteleg shrimp. Both the anterior and posterior intestine were composed with high proportions of *Oceaniovalibus*, *Streptococcus*, *Actibacter*, *Ilumatobacter*, and *Litorilinea*. On the other hand, the hepatopancreas and the stomach reported high abundance of *Salinarimonas*, *Sphingomonas*, and *Oceaniovalibus*. The genus *Oceaniovalibus* was present in all segments with notably high frequency. *Oceaniovalibus* is a marine bacterium belonging to the family *Rhodobacteraceae* [48]. The members of *Rhodobacteraceae* can reduce cold stress in whiteleg shrimp as well as drive various physiological and metabolic processes, and they exhibit probiotic potential [6]. In a previous report, *Oceaniovalibus* was found to be enriched in the intestine of whiteleg shrimp supplemented with 5-aminolevulinic acid, thus indicating a healthy microbiome composition [49]. *Streptococcus* has been used as a probiotic to inhibit *Vibrio* infection in shrimp. However, some strains of *Streptococcus* are pathogens, meaning it is necessary to evaluate their pathogenicity before formulating them as probiotics [50, 51]. The genus *Salinarimonas* was found to be strikingly high in all samples of hepatopancreas, which clearly showed the presence of a distinct set of bacterial compositions. *Salinarimonas* was also relatively high in the stomach samples when compared to the intestinal samples. Strains of *Salinarimonas* are capable of nitrate reduction and organic pollutant degradation, thus pointing to their potential utility in the bioremediation field [52, 53]. The nitrate reduction ability of *Salinarimonas* may also play a crucial role in minimizing ammonia toxicity from the hepatopancreas and stomach. There is currently limited information on the presence of *Salinarimonas* from the gut of whiteleg shrimp. Therefore, further investigations are needed to explore the significance of *Salinarimonas* in the gut of whiteleg shrimp. Specimens of the genus *Sphingomonas* have previously been found in shrimp gut, where they play key roles in aromatic compound degradation and alginate metabolisms [37]. The *Vibrio*s group, which is made up of well-known pathogens [54], has also been found in different segments of the GI tract at subordinate levels without causing diseases in shrimp. Overall, the difference in the bacterial community among various segments of the GI tract indicate that these microbiomes facilitate specific functions in each segment and potentially assist in defending against pathogens.

In conclusion, this study provides a comprehensive analysis of the bacterial communities inhabiting different segments of the GI tract in Pacific whiteleg shrimp. The microbiomes in the stomach, hepatopancreas, and anterior and posterior intestines all play crucial roles in maintaining shrimp health and aquaculture practices. This study reveals significant variations in bacterial diversity and composition among these GI segments, with the results highlighting the unique ecological niches present in each. The alpha and beta diversity analyses indicate higher bacterial diversity in the anterior intestine and lower diversity in the stomach. This variation in diversity suggests the influence of anatomical, physiological, and environmental factors within different GI segments. The dominant phylum across all segments is Proteobacteria, with stomach and hepatopancreas exhibiting higher abundance than the intestines. Spirochaetes and Fusobacteria are notably detected in the hepatopancreas and stomach, and this finding could potentially expand our understanding of microbial diversity in these regions. At the genus level, distinct bacterial profiles are observed in each GI segment: *Oceaniovalibus*, *Streptococcus*, *Actibacter*, *Ilumatobacter*, and *Litorilinea* dominate the intestines, while *Salinarimonas*, *Sphingomonas*, and *Oceaniovalibus* prevail in the stomach and the hepatopancreas. The presence of *Salinarimonas*, with its potential roles in nitrate reduction and pollutant degradation, warrants further investigation. Overall, this study helps fill in a critical knowledge gap by holistically exploring the microbial ecology in interconnected segments of the shrimp's GI tract. The findings enhance our understanding of the microbiome's role in shrimp health, immune defense, and nutrient processing. The information generated herein has implications for sustainable shrimp aquaculture practices. Future research should focus on exploring the functional roles played by specific bacterial taxa in different GI segments of the whiteleg shrimp using metagenomic and metatranscriptomic approaches. Experimental studies could also be conducted to assess the impact of these bacterial communities on shrimp health, immune function, and digestion. Investigating the ecological interactions among bacterial taxa within the GI tract may also provide valuable insights into the dynamics of microbial communities and their influence on shrimp health and aquaculture practices.

## Supplemental Materials

Supplementary data for this paper are available on-line only at http://jmb.or.kr.
