# Supplementary material for: Unveiling the Bacterial Community across the Stomach, Hepatopancreas, Anterior Intestine, and Posterior Intestine of Pacific Whiteleg Shrimp
Source: J Microbiol Biotechnol. 2024 Apr 29;34(6):1260–9. doi: 10.4014/jmb.2403.03039 (PMC11239424; doi:10.4014/jmb.2403.03039)
Supplement: Supplementary file 1 [file jmb-34-6-1260-supple.pdf]

J. Microbiol. Biotechnol. 2024. 34(6): 1260–1269
https://doi.org/10.4014/jmb.2403.03039

Supplementary Tables

Unveiling the bacterial community across the stomach, hepatopancreas, anterior intestine, and posterior intestine of Pacific Whiteleg shrimp

Dhiraj Kumar Chaudhary [1,2†], Sang-Eon Kim [1,2†], Hye-Jin Park[3], and Kyoung-Ho Kim [1,2*]

[1] Department of Microbiology, Pukyong National University, Busan 48513, Republic of Korea
[2] Division of Marine and Fisheries Life Sciences, Pukyong National University, Busan 48513, Republic of Korea
[3] Korea Institute of Ocean Science and Technology, Busan, Republic of Korea

[†]The authors equally contributed to this work

* Corresponding author e-mail: kimkh@pknu.ac.kr

Table S1. Metadata of whiteleg shrimp's microbiome study. The primers used in this study were 319F (5′-ACTCCTACGGGAGGCAGCAG-3′) and 806R (5′-GGACTACHVGGGTWTCTAAT-3′).

Samples	Organ	Barcode_F	Barcode_R	SampleType	BioSample Accession
Bac_H_LA3	Hepatopancreas	GTGGTATGGGAG	TACCGGCTTGCA	LA3	SAMN40199248
Bac_P_LA3	Posteiror_intestine	GTGGTATGGGAG	CCAGGGACTTCT	LA3	SAMN40199255
Bac_H_LA6	Hepatopancreas	ATGTCCGACCAA	CACCTTACCTTA	LA6	SAMN40199251
Bac_P_LA4	Posteiror_intestine	GAGCAACATCCT	TACCGGCTTGCA	LA4	SAMN40199256
Bac_A_LA4	Anterior_intestine	ACTTTAAGGGTG	CCAGGGACTTCT	LA4	SAMN40199242
Bac_A_LA5	Anterior_intestine	TGTTGCGTTTCT	CACCTTACCTTA	LA5	SAMN40199243
Bac_S_LA9	Stomach	TGTCTCGCAAGC	GAGACTATATGC	LA9	SAMN40199267
Bac_S_LA7	Stomach	AGGTACGCAATT	CACCTTACCTTA	LA7	SAMN40199265
Bac_P_LA9	Posteiror_intestine	GAGGAGTAAAGC	GAGACTATATGC	LA9	SAMN40199261
Bac_P_LA7	Posteiror_intestine	ACAGCCACCCAT	TTAACTGGAAGC	LA7	SAMN40199259
Bac_A_LA6	Anterior_intestine	ATGTCCGACCAA	ATAGTTAGGGCT	LA6	SAMN40199244
Bac_H_LA7	Hepatopancreas	AGGTACGCAATT	ATAGTTAGGGCT	LA7	SAMN40199252
Bac_H_LA4	Hepatopancreas	ACTTTAAGGGTG	ATCTAGTGGCAA	LA4	SAMN40199249
Bac_S_LA4	Stomach	ACTTTAAGGGTG	TACCGGCTTGCA	LA4	SAMN40199262
Bac_A_LA8	Anterior_intestine	TGTCTCGCAAGC	TTAACTGGAAGC	LA8	SAMN40199246
Bac_A_LA3	Anterior_intestine	GTGGTATGGGAG	ATCTAGTGGCAA	LA3	SAMN40199241
Bac_H_LA9	Hepatopancreas	GAGGAGTAAAGC	TTAACTGGAAGC	LA9	SAMN40199254
Bac_P_LA5	Posteiror_intestine	TGTTGCGTTTCT	ATAGTTAGGGCT	LA5	SAMN40199257
Bac_P_LA6	Posteiror_intestine	ATGTCCGACCAA	GCACTTCATTTC	LA6	SAMN40199258
Bac_H_LA5	Hepatopancreas	GAGCAACATCCT	CCAGGGACTTCT	LA5	SAMN40199250
Bac_S_LA8	Stomach	ACAGCCACCCAT	CGCGGTTACTAA	LA8	SAMN40199266
Bac_A_LA9	Anterior_intestine	GAGGAGTAAAGC	CGCGGTTACTAA	LA9	SAMN40199247
Bac_A_LA7	Anterior_intestine	AGGTACGCAATT	GCACTTCATTTC	LA7	SAMN40199245
Bac_P_LA8	Posteiror_intestine	TGTCTCGCAAGC	CGCGGTTACTAA	LA8	SAMN40199260
Bac_S_LA6	Stomach	TGTTGCGTTTCT	GCACTTCATTTC	LA6	SAMN40199264
Bac_S_LA5	Stomach	GAGCAACATCCT	ATCTAGTGGCAA	LA5	SAMN40199263
Bac_H_LA8	Hepatopancreas	ACAGCCACCCAT	GAGACTATATGC	LA8	SAMN40199253

Table S2. Alpha-diversity indexes of bacterial communities in different shrimp samples.

Gastrointestinal tract	Samples	Observed	Shannon	ACE	Chao1
Anterior_intestine	Bac_A_LA3	386.0	5.4	386.2	386.0
	Bac_A_LA4	213.0	5.0	213.0	213.0
	Bac_A_LA5	93.0	4.3	93.0	93.0
	Bac_A_LA6	330.0	5.5	330.3	330.0
	Bac_A_LA7	417.0	5.6	417.4	417.0
	Bac_A_LA8	91.0	4.2	91.0	91.0
	Bac_A_LA9	271.0	5.3	271.4	271.0
Hepatopancreas	Bac_H_LA3	98.0	3.7	98.0	98.0
	Bac_H_LA4	51.0	3.4	51.0	51.0
	Bac_H_LA5	49.0	2.9	49.0	49.0
	Bac_H_LA6	57.0	3.1	57.0	57.0
	Bac_H_LA7	229.0	4.4	230.9	230.5
	Bac_H_LA8	61.0	3.9	65.2	61.0
	Bac_H_LA9	70.0	3.9	70.0	70.0
Posterior_intestine	Bac_P_LA3	164.0	4.8	164.0	164.0
	Bac_P_LA4	142.0	4.7	142.5	142.0
	Bac_P_LA5	369.0	5.6	369.0	369.0
	Bac_P_LA6	328.0	5.3	328.5	329.0
	Bac_P_LA7	278.0	5.3	278.0	278.0
	Bac_P_LA8	211.0	5.1	211.4	211.0
	Bac_P_LA9	383.0	5.6	383.0	383.0
Stomach	Bac_S_LA4	24.0	3.0	82.0	24.0
	Bac_S_LA5	21.0	2.9	73.4	21.0
	Bac_S_LA6	78.0	4.1	78.0	78.0
	Bac_S_LA7	53.0	3.8	83.4	53.0
	Bac_S_LA8	85.0	4.2	85.0	85.0
	Bac_S_LA9	94.0	4.2	94.0	94.0

Table S3. Relative abundance of the phyla observed in anterior intestine, hepatopancreas, posterior intestine, and stoamch of whiteleg shrimp. 1, Proteobacteria; 2, Cyanobacteria; 3, Firmicutes; 4, TM7; 5, Bacteroidetes, 6, Actinobacteria; 7, Verrucomicrobia; 8, WS6; 9, Chloroflexi; 10, Planctomycetes; 11, Chlamydiae; 12, Spirochaetes; 13, Fusobacteria; 14, Tenericutes; 15, TM6; 16, Lentisphaerae; 17, OD1l; 18, Nitrospirae.

Samples	1	2	3	4	5	6	7	8	9	10	11	12	13	14	15	16	17	18
Bac_A_LA3	31.8	15.1	13.1	2.6	13.3	12.6	1.6	0.5	1.8	1.2	0.7	0.0	0.0	0.0	0.0	0.0	0.0	0.0
Bac_A_LA4	45.9	8.3	1.3	2.5	20.7	14.0	0.6	0.3	1.7	0.3	0.0	0.0	0.0	0.0	0.0	0.0	0.0	0.0
Bac_A_LA5	33.0	3.3	39.4	3.2	7.3	7.5	1.0	0.5	1.5	0.0	0.0	0.0	0.4	0.0	0.0	0.0	0.0	0.0
Bac_A_LA6	39.8	5.4	13.1	2.1	18.3	10.5	1.0	0.7	1.3	0.4	0.1	0.0	0.1	0.0	0.0	0.0	0.0	0.0
Bac_A_LA7	41.2	10.4	6.2	3.6	14.4	11.3	1.7	0.6	1.8	0.4	0.4	0.0	0.0	0.0	0.0	0.0	0.0	0.0
Bac_A_LA8	33.7	4.7	41.7	1.5	5.7	8.3	0.0	0.0	1.0	0.0	0.0	0.0	0.0	0.0	0.0	0.0	0.0	0.0
Bac_A_LA9	33.9	12.2	9.0	2.8	17.3	11.6	2.2	0.3	2.0	0.9	0.3	0.0	0.0	0.0	0.0	0.0	0.0	0.0
Bac_H_LA3	66.5	1.5	11.0	4.5	6.0	4.9	0.1	0.3	0.1	0.1	0.0	0.8	0.3	0.0	0.0	0.0	0.0	0.0
Bac_H_LA4	69.3	2.2	7.3	4.7	5.4	6.1	0.2	0.1	0.0	0.8	0.0	0.0	0.8	0.0	0.0	0.0	0.0	0.0
Bac_H_LA5	72.9	0.4	7.8	4.4	3.3	4.1	0.8	0.2	1.5	0.0	0.8	0.0	0.0	0.0	0.0	0.4	0.0	0.0
Bac_H_LA6	84.8	0.1	0.8	4.6	5.7	0.7	0.0	0.0	0.2	0.2	0.0	0.0	0.0	0.0	0.0	0.0	0.0	0.0
Bac_H_LA7	61.3	2.8	9.0	7.8	3.3	5.7	1.2	0.9	1.2	0.6	0.3	0.4	0.0	0.0	0.1	0.0	0.0	0.0
Bac_H_LA8	57.6	6.9	5.7	2.8	8.5	6.6	0.1	0.0	0.0	0.6	0.0	0.0	7.5	0.0	0.0	0.0	0.0	0.0
Bac_H_LA9	62.1	6.9	8.9	5.7	7.7	4.9	0.4	0.3	0.6	0.0	0.0	0.2	0.0	0.0	0.0	0.0	0.0	0.0
Bac_P_LA3	41.6	9.1	13.8	1.8	13.8	9.9	0.7	0.2	2.7	0.9	0.1	0.0	0.0	0.0	0.0	0.0	0.0	0.0
Bac_P_LA4	34.1	14.4	4.3	2.7	18.1	14.5	3.8	0.0	1.8	1.5	0.4	0.0	0.0	0.0	0.0	0.0	0.0	0.0
Bac_P_LA5	36.4	4.7	22.8	1.9	12.2	13.6	0.6	0.5	1.1	0.5	0.2	0.0	0.4	0.0	0.0	0.0	0.1	0.0
Bac_P_LA6	38.0	5.8	19.5	1.3	15.7	10.2	0.9	0.0	2.4	0.5	0.2	0.0	0.0	0.2	0.0	0.0	0.0	0.0
Bac_P_LA7	41.6	8.8	5.1	5.5	11.3	14.0	0.8	1.1	2.1	0.3	0.3	0.0	0.0	0.1	0.0	0.0	0.0	0.0
Bac_P_LA8	29.1	9.6	22.3	2.5	15.0	10.0	2.5	0.6	1.4	0.2	0.4	0.0	0.9	0.0	0.0	0.0	0.0	0.0
Bac_P_LA9	38.0	9.9	8.2	2.7	17.2	10.8	1.4	0.8	2.1	0.6	0.4	0.0	0.0	0.3	0.0	0.0	0.0	0.0
Bac_S_LA4	70.1	3.2	5.7	1.8	3.4	12.1	0.7	0.0	0.0	0.0	0.0	0.0	0.0	0.0	0.0	0.0	0.0	0.0
Bac_S_LA5	91.0	1.0	1.2	1.4	0.0	1.5	0.0	0.0	0.0	0.0	0.0	0.0	0.0	0.0	0.0	0.0	0.0	0.0
Bac_S_LA6	52.4	3.2	11.0	1.0	10.4	10.4	0.9	0.4	0.0	0.1	0.0	0.0	1.3	0.0	0.0	0.0	0.0	0.0
Bac_S_LA7	67.2	0.3	12.1	0.0	7.7	6.9	2.7	0.0	0.0	0.0	0.0	0.0	0.0	0.0	0.0	0.0	0.0	0.0
Bac_S_LA8	51.1	11.2	3.6	5.1	3.7	7.3	5.0	2.0	2.0	0.4	1.0	0.0	0.0	0.0	0.0	0.0	0.0	0.0
Bac_S_LA9	60.8	6.8	10.5	1.7	6.1	8.7	0.3	0.2	0.3	0.0	0.4	0.2	0.0	0.0	0.0	0.0	0.0	0.0

Table S4. Differential abunandce of major genera detected in anterior intestine, hepatopancreas, posterior intestine, and stoamch of whiteleg shrimp. The genus with greater than 1% abundance are only illustrated.

**Genera**	**Anterior_intestine**						
	**Bac_A_LA3**	**Bac_A_LA4**	**Bac_A_LA5**	**Bac_A_LA6**	**Bac_A_LA7**	**Bac_A_LA8**	**Bac_A_LA9**
Actibacter	1.3	1.9	1.4	1.8	2.2	0.0	1.8
Aliivibrio	0.0	0.0	1.2	0.0	0.0	0.0	0.0
Butyricicoccus	0.0	0.0	1.7	0.0	0.0	0.0	0.0
Clostridium	0.0	0.0	1.7	0.0	0.0	1.2	0.0
Corynebacterium	0.0	0.0	0.0	0.0	0.0	1.4	0.0
Granulicatella	0.0	0.0	0.0	0.0	0.0	1.2	0.0
Halalkalicoccus	0.0	0.0	0.0	0.0	0.0	1.0	0.0
Haloferula	1.4	0.0	0.0	0.0	1.3	0.0	1.3
Ilumatobacter	3.3	3.6	0.0	2.0	2.5	0.0	2.7
Litorilinea	1.8	1.7	1.5	1.3	1.8	0.0	2.0
Oceaniovalibus	5.2	5.9	5.5	5.2	8.2	2.0	5.3
Peptostreptococcus	0.0	0.0	0.0	0.0	0.0	1.8	0.0
Prevotella	0.0	0.0	0.0	0.0	0.0	1.2	0.0
Salinarimonas	0.0	0.0	2.1	0.0	0.0	3.4	0.0
Shimia	0.0	1.3	0.0	0.0	0.0	0.0	0.0
Sphingomonas	0.0	0.0	0.0	0.0	0.0	4.1	0.0
Streptococcus	0.0	0.0	16.5	0.0	3.2	22.0	1.1
Thioalkalivibrio	1.7	1.8	1.1	3.7	2.1	0.0	2.4
Vibrio	0.0	2.1	0.0	0.0	0.0	1.5	0.0
Yeosuana	0.0	1.1	0.0	0.0	0.0	0.0	0.0
**Genera**	**Hepatopancreas**						
	**Bac_H_LA3**	**Bac_H_LA4**	**Bac_H_LA5**	**Bac_H_LA6**	**Bac_H_LA7**	**Bac_H_LA8**	**Bac_H_LA9**
Acinetobacter	0.0	1.7	0.0	0.0	0.0	0.0	0.0
Actibacter	0.0	0.0	0.0	0.0	0.0	3.7	0.0
Aliivibrio	0.0	0.0	1.6	0.0	0.0	1.4	0.0
Butyricicoccus	0.0	0.0	0.0	0.0	1.3	0.0	0.0
Corynebacterium	0.0	0.0	0.0	0.0	0.0	1.7	0.0
Haloferula	0.0	0.0	0.0	0.0	1.0	0.0	0.0
Ilumatobacter	0.0	1.3	0.0	0.0	0.0	0.0	0.0
Isobaculum	1.5	0.0	0.0	0.0	0.0	0.0	1.3
Kriegella	0.0	0.0	0.0	0.0	0.0	1.1	0.0
Litorilinea	0.0	0.0	1.5	0.0	1.2	0.0	0.0

Nocardioides	0.0	0.0	0.0	0.0	0.0	1.3	0.0
Oceaniovalibus	1.7	4.9	0.0	0.0	3.1	4.4	5.5
Peptostreptococcus	0.0	0.0	0.0	0.0	0.0	0.0	0.0
Psychrilyobacter	0.0	0.0	0.0	0.0	0.0	7.5	0.0
Salinarimonas	40.7	37.6	56.8	67.8	37.0	6.0	19.6
Sphingomonas	2.4	2.1	2.4	1.5	0.0	2.1	8.2
Staphylococcus	0.0	1.7	0.0	0.0	0.0	0.0	0.0
Streptococcus	1.1	3.0	0.0	0.0	1.8	2.8	2.4
Vibrio	1.0	1.5	2.2	0.0	0.0	3.6	1.2
**Genera**	**Posterior_intestine**						
	**Bac_P_LA3**	**Bac_P_LA4**	**Bac_P_LA5**	**Bac_P_LA6**	**Bac_P_LA7**	**Bac_P_LA8**	**Bac_P_LA9**
Actibacter	1.4	2.8	1.7	1.5	1.5	1.1	2.4
Bacteroides	0.0	0.0	0.0	0.0	0.0	1.7	0.0
Clostridium	0.0	0.0	1.7	0.0	0.0	0.0	0.0
Corynebacterium	0.0	0.0	1.3	0.0	0.0	0.0	0.0
Haloferula	0.0	2.5	0.0	0.0	0.0	2.0	1.1
Ilumatobacter	2.4	3.9	2.6	2.0	3.9	1.7	2.4
Litorilinea	2.7	1.8	1.1	2.4	2.1	1.4	2.1
Nocardioides	1.0	0.0	0.0	0.0	0.0	0.0	0.0
Oceaniovalibus	6.4	4.8	5.8	5.4	10.2	5.4	6.8
Pseudozobellia	0.0	0.0	0.0	0.0	0.0	0.0	1.2
Roseibacillus	0.0	1.2	0.0	0.0	0.0	0.0	0.0
Salinarimonas	3.7	1.8	0.0	0.0	0.0	0.0	0.0
Shimia	1.1	0.0	0.0	0.0	0.0	0.0	0.0
Streptococcus	0.0	0.0	10.7	0.0	2.9	8.3	1.2
Thioalkalivibrio	2.4	0.0	2.4	2.9	2.2	0.0	2.3
Vibrio	2.6	0.0	0.0	2.0	0.0	3.4	0.0

**Genera**	**Stomach**					
	**Bac_S_LA4**	**Bac_S_LA5**	**Bac_S_LA6**	**Bac_S_LA7**	**Bac_S_LA8**	**Bac_S_LA9**
Actibacter	2.6	0.0	1.1	1.1	0.0	0.0
Albidovulum	0.0	0.0	1.3	0.0	0.0	0.0
Aliivibrio	0.0	0.0	1.1	0.0	0.0	0.0
Alkanindiges	0.0	0.0	1.5	0.0	0.0	0.0
Brevundimonas	0.0	0.0	0.0	1.1	0.0	0.0
Corynebacterium	7.4	0.0	0.0	0.0	0.0	0.0
Haloferula	0.0	0.0	0.0	2.7	5.0	0.0
Hoeflea	0.0	0.0	0.0	0.0	1.2	0.0

Ilumatobacter	0.0	0.0	3.8	2.0	0.0	1.6
Kriegella	0.0	0.0	0.0	0.0	0.0	1.2
Litorilinea	0.0	0.0	0.0	0.0	2.0	0.0
Maribius	0.0	0.0	1.1	0.0	0.0	0.0
Oceaniovalibus	9.1	4.6	5.8	2.5	18.7	2.2
Salinarimonas	5.1	8.0	4.4	4.8	0.0	20.7
Salipiger	2.3	0.0	0.0	2.3	0.0	0.0
Sphingomonas	4.4	12.1	1.7	4.7	1.4	4.4
Staphylococcus	1.1	0.0	1.0	0.0	0.0	0.0
Streptococcus	3.0	0.0	1.8	2.2	0.0	3.0
Thioalkalivibrio	0.0	0.0	1.5	0.0	0.0	1.4
Vibrio	2.6	0.0	0.0	0.0	0.0	2.0

Table S5. Correlation values estimated during correlation network analysis by Sparse Estimation of Correlations Among Microbiomes (SECOM) method.

**Taxon-1**	**Taxon2**	**Correlation value**	***p*-value**
TM7	Proteobacteria	0.6576	0.0004
Proteobacteria	TM7	0.6576	0.0004
Firmicutes	Actinobacteria	0.4222	0.0282
Actinobacteria	Firmicutes	0.4222	0.0282
WS6	TM7	0.3892	0.1104
TM7	WS6	0.3892	0.1104
Firmicutes	Cyanobacteria	0.3077	0.1184
Cyanobacteria	Firmicutes	0.3077	0.1184
Firmicutes	Bacteroidetes	0.3073	0.1267
Bacteroidetes	Firmicutes	0.3073	0.1267
Firmicutes	Chloroflexi	0.2360	0.3308
Chloroflexi	Firmicutes	0.2360	0.3308
Unclassified	TM7	0.1888	0.3661
TM7	Unclassified	0.1888	0.3661
WS6	Proteobacteria	0.1366	0.5890
Proteobacteria	WS6	0.1366	0.5890
Verrucomicrobia	Firmicutes	0.1137	0.6431
Firmicutes	Verrucomicrobia	0.1137	0.6431
WS6	Firmicutes	-0.0734	0.7723
Firmicutes	WS6	-0.0734	0.7723
TM7	Chloroflexi	-0.0742	0.7629
Chloroflexi	TM7	-0.0742	0.7629
Verrucomicrobia	TM7	-0.1883	0.4543
TM7	Verrucomicrobia	-0.1883	0.4543
Proteobacteria	Bacteroidetes	-0.2377	0.2424
Bacteroidetes	Proteobacteria	-0.2377	0.2424
TM7	Bacteroidetes	-0.2407	0.2572
Bacteroidetes	TM7	-0.2407	0.2572
TM7	Cyanobacteria	-0.3080	0.1341
Cyanobacteria	TM7	-0.3080	0.1341
Proteobacteria	Firmicutes	-0.3511	0.0725
Firmicutes	Proteobacteria	-0.3511	0.0725
TM7	Actinobacteria	-0.3723	0.0668
Actinobacteria	TM7	-0.3723	0.0668
TM7	Firmicutes	-0.4371	0.0289
Firmicutes	TM7	-0.4371	0.0289
Verrucomicrobia	Proteobacteria	-0.5102	0.0256
Proteobacteria	Verrucomicrobia	-0.5102	0.0256
Proteobacteria	Chloroflexi	-0.5111	0.0253
Chloroflexi	Proteobacteria	-0.5111	0.0253
Proteobacteria	Actinobacteria	-0.5611	0.0023
Actinobacteria	Proteobacteria	-0.5611	0.0023
Proteobacteria	Cyanobacteria	-0.5927	0.0011
Cyanobacteria	Proteobacteria	-0.5927	0.0011
